# Reverse Shoulder Arthroplasty, Deltopectoral Approach vs. Anterosuperior Approach: An Overview of the Literature

**DOI:** 10.3389/fsurg.2021.721054

**Published:** 2021-11-18

**Authors:** Paraskevas Georgoulas, Aliki Fiska, Athanasios Ververidis, Georgios I. Drosos, Evanthia Perikleous, Konstantinos Tilkeridis

**Affiliations:** ^1^Department of Orthopedic Surgery, Medical School, Democritus University of Thrace, University General Hospital of Alexandroupolis, Alexandroupolis, Greece; ^2^Department of Anatomy, Medical School, Democritus University of Thrace, University General Hospital of Alexandroupolis, Alexandroupolis, Greece; ^3^Medical School, Democritus University of Thrace, Alexandroupolis, Greece

**Keywords:** reverse shoulder arthroplasty, deltopectoral approach, anterosuperior approach, notching, cuff tear arthropathy

## Abstract

Reverse shoulder arthroplasty (RSA) has become an optimal treatment for numerous orthopedic entities, such as rotator cuff tear arthropathies, pseudoparalysis, fracture sequelae, acute fractures, failed arthroplasties, osteoarthritis, and rheumatoid arthritis, and is linked with relief of topical pain and regaining of functionality. Presently, RSA has been conducted through anterosuperior (AS) or deltopectoral (DP) approach. The aim of the study was to discuss both approaches and to examine broadly their features to render a comparison in terms of clinical effectiveness. An electronic search in PubMed, EMBASE, and Google Scholar databases was performed, using combinations of the following keywords: RSA, DP approach, AS approach, notching, and cuff tear arthropathy. A total of 61 studies were found, and 16 relevant articles were eventually included. Currently published literature has not shown significant diversities in the clinical course due to approach preference; risk of instability seems to be greater in DP approach, while regarding scapular notching and fracture rates the findings were conflicted. In addition, the AS approach has been associated with decreased risk of acromial and scapular spine fractures. In conclusion, both surgical approaches have shown similar clinical outcomes and effectiveness concerning pain and restoring range of motion (ROM) in rotator cuff tear arthropathies. In the future, further investigations based on large-scale well-designed studies are required to address clinical gaps allowing in-depth comparison of both approaches.

## Introduction

Osteoarthritis is an ordinary degenerative entity of the shoulder joint, signalized by compressed glenohumeral joint space, and the most favorable therapy is shoulder replacement (1). Fundamentally, shoulder osteoarthritis displays regional pain, rigidity, and restriction of shoulder function. Consequently, shoulder pain is correlated with a remarkable disability, disability assertions, augmented usage of healthcare resources, and marked morbidity, principally in the elderlies (2).

Surgical treatment is commonly performed in individuals in whom conservative therapy was unsuccessful as they continued to experience persistent symptoms associated with impaired quality of life. Nevertheless, the results of anatomical total shoulder arthroplasty in cases with rotator cuff tear arthropathies have been scarce (3, 4); reverse shoulder arthroplasty (RSA) may be an appropriate, effective open procedure (5, 6). RSA has become one of the most significant medical progresses in the field of shoulder arthroplasty, in the last three decades and from its genesis has achieved outstanding popularity in view of the capability to treat patients with severe rotator cuff deficiency.

Taking a step back in time, in the 1970s Beddow and Alloy had conducted a pioneering RSA in patients with rheumatoid arthritis; however, they did not publish the extracted outcomes. Finally, in 1987 Grammont et al. proposed RSA to cure rotator cuff tear arthropathy. In spite of the promising initial outcomes of European cases, RSA was eventually approved by the US Food and Drug Administration not earlier than 2004 (7), and since then the quantity of RSAs, carried out annually, is rising sharply (8). Nowadays, the procedure indications have been expanded to a great degree, such as fracture sequelae, acute fractures, massive cuff tears, salvage revision arthroplasty, osteoarthritis, and rheumatoid arthritis (9–12).

Previously, transacromial approaches, reported by Grammont, were unsuccessful due to the failure of acromial fixation (13, 14); subsequently, at present, they are not so much popular. Currently, the two commonest surgical approaches are the anterosuperior (AS) and the deltopectoral (DP) approaches (15). Although, the majority of orthopedics prefer the DP approach, in 1993 Mackenzie delineated a novel procedure, the AS approach (16). Both approaches can provide analogous initial clinical outcomes, allowing the secure and reproducible revelation of the glenoid and humerus (15). The decision-making task, considering the most suitable approach, must count a complex combination of many elements, comprising surgeon predilection and patient-oriented aspects.

Momentarily, few published data compare the advantages, disadvantages, and clinical courses following the DP or AS approach in RSA. Herein, we execute a technical overview of the ongoing data respecting both approaches in the context of shoulder arthroplasty. We aim to describe both surgical approaches shedding new light on their comparison, analyzing features, such as clinical outcomes, efficacy, and long-term effects.

## Search Strategy

We performed a comprehensive review of the medical literature using the following databases: PubMed, EMBASE, and Google Scholar, using combinations of the following keywords: RSA, DP approach, AS approach, notching, and cuff tear arthropathy. Articles were screened by title, abstract, and full text to locate all manuscripts pertinent to this topic. The search included all types of articles written in English until May 2021.

**Figure 1:**
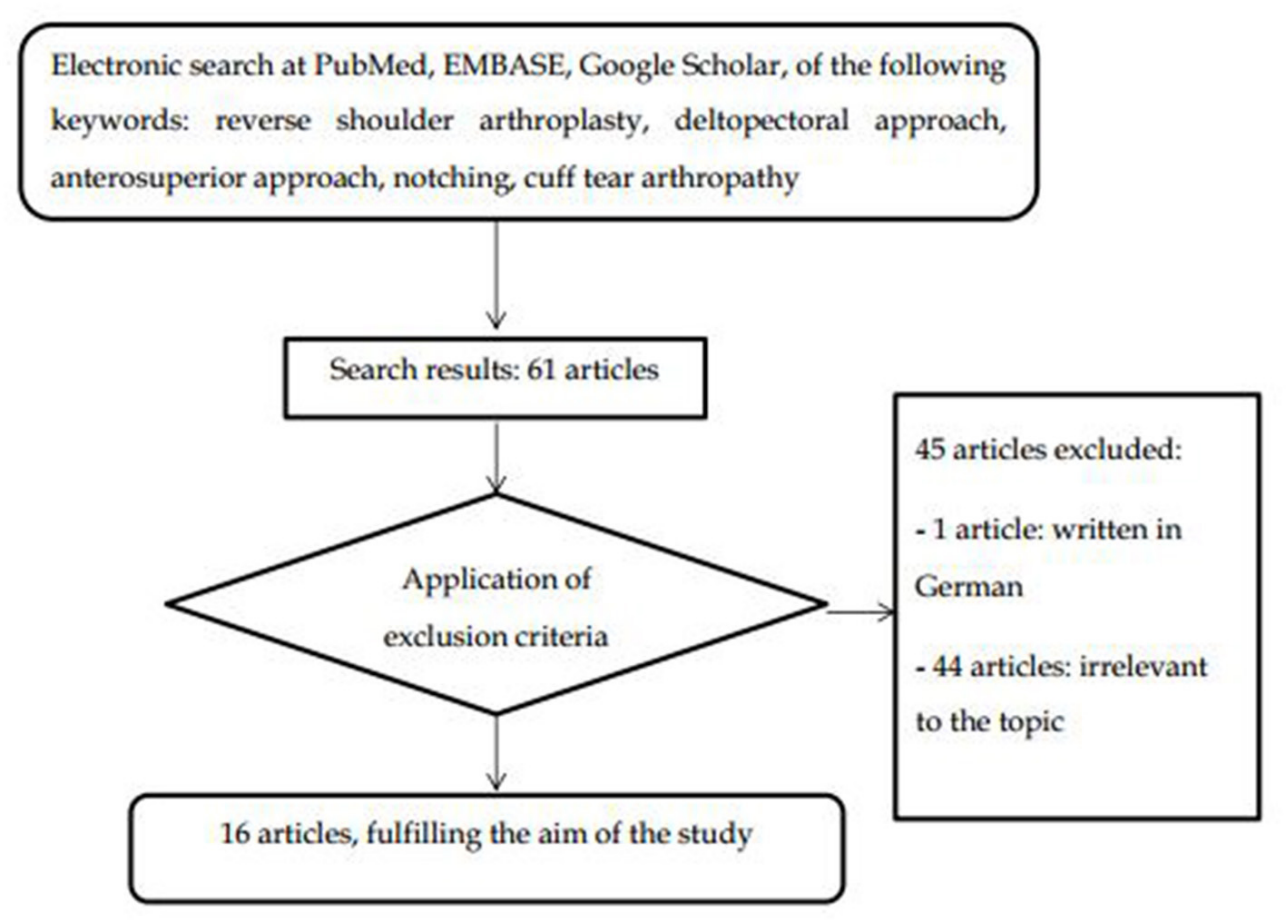
Flow chart of the search strategy.

A total of 61 studies were found, and 16 articles were finally included in the present review. Among them, the vast majority were retrospective studies (*n* = 11), a few were prospective studies (*n* = 2), case reports (*n* = 2), and comparative reviews (*n* = 2, one of which was a combination of retrospective study with a comparative review of the literature). Exclusion criteria were the following: not written in English (*n* = 1, German), not relevant data (*n* = 44), for example, cadaveric studies and extraneous issues, such as studies related to unusual anatomic variations, comparative studies among DP or AS with other approaches, and so on. The followed strategy is demonstrated in Figure 1.

## Surgical Approaches

### DP Approach

The DP approach has been delineated in former studies (10, 17). Firstly, we place the patient in a beach chair position, leaving anterior and posterior shoulder parts free of obstacles. The operation should be initiated by locating bony landmarks of the shoulder, such as acromion, clavicle, and coracoid process. An ~10 cm oblique skin section is conducted in the anterior side of the shoulder, starting from coracoid apophysis to the deltoid muscle, near the DP groove. Then, the cephalic vein is recognized and, commonly, is withdrawn laterally. The subacromial adhesions are unleashed, and the clavipectoral aponeurosis is cutted out. Afterward, the biceps tendon is being determined and subsequently tenotomized or tenodesed. The subscapularis is released from the lesser tubercle, letting a tissue segment for reconstruction. The bursa should be free from the humerus. Following humerus preparation, the glenoid is revealed and prepared. The advantages and disadvantages of this procedure are presented in Table 1 (15, 18–22).

**Table 1:** Advantages and disadvantages of surgical approaches.

**Surgical approach**	**Advantages**	**Disadvantages**
Deltopectoral (15, 18–21)	- Maintain deltoid and pectoralis origin - Access across the muscles - Reduce risk of denervation - Minor bleeding - Capable to be extended - Unobstructed approach to humerus - Effortless access in inferior structures, such as inferior capsule - Inferior part of the glenoid easily accessible	- High risk of instability - Inconvenience in approaching posterior structures - Nerve injury - Baseplate being set in an inappropriate position in RSA - Risk of acromial and scapular spine fractures
Anterosuperior (15, 18, 21, 22)	- Direct exposure of glenoid cavity - Restricts risk of glenohumeral instability - It preserves the subscapularis muscle - Baseplate positioning in the AP direction - Unimpeded access of posterior part of glenoid	- Debilitates the deltoid muscle through mechanical or neurologic detriments - Poor access to inferior portion of glenoid cavity - Positioning of glenoid baseplate in neutral or inferiorly tilted place is complicated - No extension permitted - Risk of scapular spine fracture

*RSA, reverse shoulder arthroplasty; AP, anteroposterior*.

### AS Approach

The AS approach has been reported by Molé et al. (22). The patient takes the beach chair position, keeping the anterior and posterior parts of the shoulder joint without any obstacles (22). The approach must be started by finding the location of bony landmarks around the shoulder, comprising anterior and posterior acromion sides, anterior clavicle part, and acromioclavicular joint. Either a longitudinal or a horizontal skin incision is utilized, oriented on a mark posterior to the anterolateral edge of the acromioclavicular joint. The deltoid muscle is divided among anterior and middle thirds, and this continues until 5 cm from the acromion to avoid axillary nerve injury. The surgeon detaches the deltoid muscle from the anterior surface of the acromion and removes the coracoacromial ligament in a single layer. An acromioplasty may be implemented; however, it is better to be sidestepped so that it does not debilitate the acromion. The intra-articular segment of the biceps tendon, if exist, must be tenotomized. The insertion of the subscapularis is maintained in every single case. To place humeral prosthesis, manipulations on the distal humerus while the extremity in extension permits the subluxation of the humeral head. The revelation of glenoid is integrated with excision of the labrum and capsular releasement of the inferior 50% of the glenoid (23). The advantages and disadvantages of this approach are displayed in Table 1 (15, 18–22).

## Accumulated Results from the Literature

The total number of shoulders, from the reviewed literature, was 1,970 and involved 1,941 patients (14, 15, 22–35). Among a total of 1,970 RSAs, 719 were AS and 1,251 DP approaches, percentages equals to 36.5 and 63.5%, respectively (14, 15, 22–35). Although the DP approach was overall observed to a greater extent, it seems that the AS approach has been used mainly in European countries (14, 22, 25, 26, 32, 34, 35), especially in France (14, 22, 25, 26) and in some USA centers (15, 23). Irrespective of the RSA indications, positive outcomes have been described either with AS or DP approach. None of the published studies has shown considerable dissimilarities in the clinical course due to surgical approach preference. Regardless, an emerging body of studies pointed out variations in material placing, scapular notching, and intraoperative and postoperative outcomes. Table 2 summarizes the current literature (14, 15, 22–35).

**Table 2:** Summary of studies assessing deltopectoral and anterosuperior approaches in RSA.

**Study**	**Type of study**	**Participants**	**Length of follow-up**	**Main results**	**Comments**
Lädermann et al. (14)	**-** Original article, retrospective multicentric study	**-** 144 RSAs in 142 cases; 109 were DP and 35 AS approach	**-** At least 1 year, mean time for AS 19.7 months and 18.3 months for DP	**-** The humeral cut by the AS approach was lesser	**-** Divergence in the cut was partly corrected by the usage of thicker polyethylene import
Gillespie et al. (15)	**-** Retrospective study and comparative literature review	**-** 93 cases, 62 underwent AS and 31 DP approach	**-** A minimum of 2 years, average time was 3.1 years	**-** No significant differences in postoperative range of motion, scapular notching, and position of glenoid baseplate **-** A DP case experienced an anterior dislocation **-** 3 AS cases had a deltoid dehiscence	**-** Commonly exposure of proximal humerus is less extensive in AS approach **-** Longer-term follow-up is required
Molé et al. (22)	**-** Comparative review	**-** 527 RSAs, 300 were implanted using DP and 227 AS	**-** At least 2 years	**-** Risk of instability appears greater in DP approach **-** Similar Constant-Murley score **-** Scapular notching arised in 74% of AS approach Vs 63% of DP approach **-** Fracture rates were similar, 5.6% in DP Vs 2.2% in AS approach **-** Loosening appears in 4.8% of AS Vs 2.3% of DP approach	**-** Postoperative axillary paresis occurs uncommon **-** No tears in the deltoid muscle attachments **-** Acromial and humeral fractures represented the majority of fracture cases **-** Loosening tend to linked with superior tilt of the glenoid implant
Aibinder et al. (23)	**-** Original article, retrospective study	**-** 109 cases underwent RSAs, 87 had AS approach and 22 DP	**-** A minimum of 2 years, mean time for AS 3.9 years and 2.8 years for DP	**-** 1 dislocation in the AS group **-** Glenoid inclination, range of motion, and pain scores had no statistically significant difference among approaches **-** Similar rates of scapular notching	**-** Absence of statistically significant difference in baseplate tilt or position between the approaches
Whatley et al. (24)	**-** Original article, retrospective study	**-** 199 RSAs, all utilizing DP approach	**-** All >12 months	**-** 3/199 had a rupture of deltoid muscle	**-** All 3 had a history of a rotator cuff restoration with a mini-open approach
Lévigne et al. (25)	**-** Original article, retrospective study	**-** 326 patients, 337 shoulders; a total of 267 cases underwent DP and 70 cases AS	**-** A minimum of 1 year, average time was 47 months	**-** AS approach was related with notching at 86% and DP at 56%**-** Etiology determined the rate of notching	**-** Notching occurred commonly in cuff tear arthropathy **-** Positioning of baseplate affects scapular notching; high positioning and superior tilting should be avoided
Melis et al. (26)	**-** Original article, retrospective study	**-** 68 RSAs in 65 patients; in 30 shoulders DP was performed and in 38 AS	**-** A minimum of 8 years, mean time was 9.6 years	**-** Scapular notch was occurred in 60 shoulders and was linked with AS approach	**-** In the mean follow-up period they did not detect glenoid loosening linked with scapular notching
Kim et al. (27)	**-** Case report	**-** A 64-year-old woman underwent DP approach	**-** >30 months, fracture found on follow-up radiographs at 6th postoperative week	**-** She experienced a non-traumatic clavicle fracture	**-** Clavicle fracture could present as a result of stress initiating from clavicle during surgery, or as a result of extra tensioning of deltoid during RSA
Edwards et al. (28)	**-** Original article, prospective study	**-** 138 RSAs through DP approach	**-** A minimum of 21 months, average time was 36 months	**-** Subscapularis was reparable in 62/138 cases and irreparable in 76/138; 7 dislocations were presented	**-** All dislocations were in cases with irreparable subscapularis
Pizzo et al. (29)	**-** Case report	**-** A 73-year-old female underwent DP approach	**-** At 4th month had normal nerve function	**-** An unusual anatomic variant of axillary nerve was found, specifically it was deep to the cephalic vein inside DP interval	**-** Maintenance of the axillary nerve is of great significance, as it provides motor innervation to the deltoid muscle
Clark et al. (30)	**-** Original article, retrospective study	**-** 120 RSAs, in 111 patients, through DP approach	**-** A minimum of 6 months, and ranged up to 62 months	**-** 55/120 underwent DP approach without subscapularis repair, 65/120 with subscapularis repair- Dislocation presented in 3 shoulders in non-repair group and 2 cases in repair group	**-** Among the two groups no significant effect on the risk of complication rate, dislocation events, infection, disassociation, or function was occurred
Al-Hadithy et al. (31)	**-** Original article, retrospective study	**-** 41 RSAs, 37 patients, all were AS approach	**-** A minimum of 20 months, mean time was 5 years	**-** 28/41 occurred with scapular notching **-** 2 cases had misplaced screws on primary x-rays **-** None early postoperative dislocations	**-** High frequency of scapular notching does not appear to have an impact on functional outcomes
Jehan et al. (32)	**-** Original article, retrospective study	**-** 46 cases, all underwent AS approach	**-** A minimum of 2 years, mean time was 49 months	**-** 4/46 patients experienced complications; 3/4 had a reoperation **-** Complications were pulmonary embolism (1/4), dissociation of glenosphere from the metaglene(1/4), deltoid detachment (1/4), and stitch abscess (1/4)	**-** None case of loosening, dislocation, and nerve damage **-** AS approach permits inferior positioning of metaglene, which is advisable in order to obstruct glenoid notching
Iacobellis et al. (33)	**-** Original article, retrospective study	**-** 33 patients underwent DP approach	**-** A minimum of 10 months, mean time was 42.3 months	**-** 4 intra-operative complications; 3 fractures, and 1 subclavian arteriorrhexis **-** 1 postoperative complication, a dislocation of the prosthesis **-** 8 cases of scapular notching	**-** All cases had proximal humeral fractures **-** DP approach is appropriate for complex proximal humeral fractures in elderly patients
Kadum et al. (34)	**-** Original article, retrospective study	**-** 56 patients underwent AS approach	**-** A minimum of 9 months, mean time was 14 months	**-** None had scapular notching **-** Absence of axillary nerve injury	**-** Follow-up period was short and further investigations are needed
Seebauer et al. (35)	**-** Original article, prospective study	**-** 57 RSAs through AS approach	**-** A minimum of 3 months, average time was 18.2 months	**-** Average Constant Score was 94% **-** Grade 1 and 2 inferior glenoid notching was shown, but neverextending or exceeding grade 3 or 4	**-** No cases of glenoid base loosening **-** All functional parameters were normal adjusted to patient's age

*RSA, reverse shoulder arthroplasty; AS, anterosuperior; DP, deltopectoral*.

Table 3 presents a synopsis of the main adverse events emanating from the literature review. Main characteristics of included studies are as follows:

- Risk of instability: seems to be greater in the DP approach (22, 28)
- Scapular notching: In some studies, AS seems to have a higher amount of this adverse outcome (22, 25, 26, 31), however, in other studies, the percentages were similar among approaches (15, 23)
- Fracture rates and other postoperative complications: Humeral fractures rate appear alike in both methods, whereas fractures of acromion and scapular spine occurred more commonly in the DP approach (22). Traumatic interventions in the deltoid muscle, such as ruptures, have been reported in both approaches (15, 24)
- Range of motion (ROM): No significant differences were observed between AS and DP approaches in respect of postoperative ROM (15, 23). Moreover, studies using either DP (27, 28, 33) or AS (31, 35) approach have well outcomes considering ROM. While, in another study preoperative and postoperative ROM, after DP approach, showed no difference in the gains concerning active forward flexion, external rotation, and internal rotation (30).

**Table 3:** Main adverse outcomes following deltopectoral and anterosuperior approaches.

**Adverse outcome**	**Studies**	**Number of RSAs (n)**	**Range (%)**
Dislocation of the prosthesis	(15, 22, 23, 26, 28, 30–35)	1,288, 1,272 cases	DP 0–5.1%, average 3.43% AS 0–3.57%, average 0.79%
Scapular notching	(15, 22, 23, 25, 26, 31, 33–35)	1,321, 1,303 cases	DP 24.24–72.4%, average 50.22% AS 0–86%, average 42.61%
Stress fractures	(15, 22, 26, 27, 30, 31)	850, 834 cases	DP 0–6.2%, average 3.45% AS 1.61–2.6%, average 2.22%
Intraoperative fractures	(33, 34)	89, 89 cases	DP 9.1% (one study) AS 1.79% (one study)

*RSA, reverse shoulder arthroplasty; DP, deltopectoral; AS, anterosuperior*.

## Discussion

The aim of this review was to comprehensively describe DP and AS approaches to compare their clinical effectiveness as revealed by published data. In the past two decades, the vast majority of RSAs has been predominantly executed using either DP or AS approach. Among a total of 1,970 shoulders concerning 1,941 patients, the main finding was that regarding clinical postoperative course none of the included studies has displayed important differences related to clinical outcomes in either DP or AS surgical approach.

Dislocation following RSA is one of the major adverse effects, and it has been reported in many follow-up studies, with percentages up to 9% (30). In our review, dislocation of the prosthesis was studied in a total of 1,288 RSAs (15, 22, 23, 26, 28, 30–35), among them 52.33% were DP approaches and 47.67% were AS approaches. As shown in Table 3, the average percentage of shoulder dislocation in DP approach was equal to 3.43% and in AS approach was 0.79%. In the study of Melis et al. four prosthetic instabilities, from a total of 68 RSAs, occurred in first postoperative month, however, the authors did not report in which one of the two approaches (26); accordingly, we could not include them in percentages calculation (Table 3).

In the study of Molé et al. 527 RSAs with a minimum 2-year follow-up were included and the risk of instability showed to be higher in the DP approach in primary and revision RSA compared to the AS approach (*P* < 0.001) (22). In the study of Edwards et al. 138 RSAs through DP approach were performed by the same surgeon and seven dislocations were reported; all had an irreparable subscapularis tendon at the time of operation (28). In another study from the USA of overall 93 cases, only in one DP case, an anterior dislocation was presented although subscapularis was repaired during the procedure (15). Likewise, in a single-center study using data of three surgeons, 120 RSAs in 111 patients were conducted using the DP approach with or without subscapularis repair, in 65 and 55 shoulders respectively; in the non-repair group 3 dislocations were identified and 2 in the repair group (30). Besides, in an Italian study, 33 individuals had undergone the DP approach and in one case, an implant dislocation was observed 2 days postoperatively and was treated surgically (33). On the other hand, in the study of Aibinder et al. 109 cases were included, among them AS approach was conducted in 87 shoulders and the DP approach in 22 shoulders; a single dislocation in the AS group and none in the DP group were reported (23). Additionally, in a UK study of 46 cases which had undergone AS approach, an individual had a dissociation between the glenosphere and the metaglene, 6 months postoperatively (32).

Scapular notching is a radiographic finding, commonly occurring after RTAs, the term describes an inferior scapular neck erosive injury due to the impaction of the humeral implant during shoulder adduction. In the present study, scapular notching was estimated in a total of 1,321 RSAs (15, 22, 23, 25, 26, 31, 33–35), among them 51.7% underwent the DP approach and 48.3% AS approach. As exhibited in Table 3, the average percentage of scapular notching in DP approach was 50.22% and in AS approach was 42.61%. In the study of Melis et al. 60 out of 68 RSAs manifest scapular notching, at a minimum follow-up of 8 years (26). However, similarly to shoulder dislocation, they did not state in which one of the two approaches was presented (26); therefore, we did not include them in percentages calculation (Table 3).

In the study of Molé et al. scapular notching was presented in 74% of AS approach group and 63% of the DP approach group; nevertheless, these small differences were not statistically significant and could be attributed to a longer follow-up period of AS approach patients (22). Nonetheless, in the study of Lévigne et al. a total of 337 shoulders had undergone RSAs and scapular notching arised in 86% of AS approach patients, in contrast, solely 56% of DP approach patients developed notching (*P* < 0.0001) (25). In a French multicenter study of 68 RSAs, with a mean follow-up period of 9.6 years, Melis et al. showed that scapular notching was occurred in 60 shoulders and was linked with AS approach (26). Moreover, in the study of Al-Hadithy et al. high rate of scapular notching was observed, specifically 28 out of 41 AS had scapular notching; although the authors declare that notching did not seem to have any impact on functional outcomes (31). In some studies no significant differences were shown in notching postoperative scores of scapular among approaches (15, 22, 23).

The clinical importance of notching is foggy as it may lead to deterioration of functional results through the reduction in shoulder flexion, abduction, and force; whereas, currently the most advantageous management of notching remains unknown. The incidence and severity of notching are associated to implant design and selected surgical technique (36). Presently, there is limited data addressing the treatment of scapular notching, and further investigations based on large well-designed studies are required to clarify successful management strategies.

Regarding our study, stress fractures were studied in a total of 850 RSAs (15, 22, 26, 27, 30, 31), among them, 56.71% were DP approaches and 43.29% were AS approaches. As indicated in Table 3, the average percentage of stress fractures in the DP approach was 3.45% and in AS approach was 2.22%. In the study of Melis et al. 2 cases out of 68 RSAs, at 1 and 9 years, respectively, after RSA, had a humeral fracture, and 1 case had an acromial fracture, 2 years postoperatively, but the authors did not report in which one of the two approaches (26); hence, we could not include them in percentages calculation (Table 3). Concerning intraoperative fractures, we only have data extracted from two studies, specifically there were three diaphyseal intraoperative fractures in 33 cases which underwent the DP approach (33), and one intraoperative fracture of the glenoid cavity in a total of 56 patients who underwent AS approach (34).

Anterosuperior approach has been associated with decreased risk of acromial and scapular spine fractures (22, 36). During DP approach, surgeons are retracting deltoid muscle, specifically they elevate the anterior one-third of the deltoid insertion, and the baseplate can be placed in an anterior or anteverted position (15) due to difficulty in approaching from an anterior side. Conversely, AS approach provides better visualization of the glenoid surface (15, 21), and the baseplate position is in the anteroposterior direction and, also, preserves the anterior soft tissue structures; presumably, leading to the lower necessity of arm lengthening (15). Thus, biomechanically, we can speculate that AS approach could probably restrict excessive load to the deltoid muscle and stress fractures at various locations along the acromion and scapular spine. Vice versa, in the DP approach longer arm length and substantial deltoid tension take place, leading to greater deltoid tension.

Patients who underwent over-tensioned RSAs can be vulnerable to acromial stress fractures, while patients who experienced under-tensioned RSAs can be prompted to dislocation (37). To our knowledge, there are no comparative studies addressing the adverse outcomes in AS vs. DP approach. Understanding of forces in the glenohumeral joint through comparative studies between the two approaches is crucial for shoulder functionality targeting in the improvement of analytical biomechanical models of the shoulder.

In the study of Iacobellis et al. 33 patients underwent the DP approach and 3 had diaphyseal fractures (33). In a case report a woman, 64-year-old, presented with a non-traumatic clavicle fracture at sixth postoperative week, following the DP approach (27). Computed tomography scanning could be supportive to estimate impingement originated from bone or heterotopic ossification, assess implant loosening, tilt, and recognize acromial fractures (38). Acromial fractures continue to be challenging for the orthopedic surgeon in terms of diagnosis and prevention; prompt diagnosis requires high clinical suspicion and implication of prevention strategies is warranted. Characteristic examples of strategies for preventing acromial fractures comprise accurate glenoid baseplate screw length and position, and circumvention of extra deltoid forces (39).

Reverse shoulder arthroplasty is linked with excellent relief of regional pain and regaining of functionality. In the study of Molé et al. no differences in terms of pain, using a 100-point rating pain scale named Constant-Murley score, were done postoperatively and did not show any difference between approaches (22). In the study of Aibinder et al. pain scores improved similarly with no statistical significance, in both approaches (23).

Furthermore, traumatic involvements of the deltoid muscle, such as ruptures, have been described in both approaches (15, 24). In the study of Gillespie et al. 3 patients among a total of 62 who underwent AS approach, eventually, had deltoid dehiscence and all needed additional treatment (15). The fact that AS approach demands detachment and restoration of the anterior deltoid may increase the risk of deltoid dehiscence or weakening. In another study, 3 cases, in a group of 199 patients, without any history of trauma were reported to have a postoperative rupture of deltoid muscle following the DP approach (24).

Reverse shoulder arthroplasty, for half a century, has been employed, with favorable outcomes in terms of restoring painless ROM in individuals with rotator cuff deficiency (5, 6). No significant differences were revealed between AS and DP approaches regarding postoperative ROM (15, 23). Additionally, studies using either DP (27, 28, 33) or AS (31, 35) approach have good outcomes considering ROM. Although in the study of Al-Hadithy et al. following AS approach, they noted impairment in functional scores and ROM after 2 years to final follow-up visit (31). However, in another study that reported preoperative and postoperative ROM after the DP approach in patients with and without repair of the subscapularis, no differences were found between these groups in the gains in active forward flexion, external rotation, and internal rotation (30).

Our review is subject to some limitations; therefore, the acknowledgment should be given for the purpose of interpretation of the aforementioned main findings. Firstly, included studies have a number of methodological issues, for instance, some have small sample sizes and short follow-up periods. Secondly, we described data from published research, comprising heterogeneous studies that did not analyze the same variables. Thirdly, regarding postoperative complications, a number of issues emerge with the most important being the subjective human factor, predominantly comfort zone of the surgeon. Fourthly, included studies were published in a time period expanding in ~15 years and during this period of time, RSA underwent deep transformations in design and surgical techniques, for instance, osteotomy of the acromion, which was a common procedure in the early years of AS approach, it is not mentioned in any of the included studies. Accordingly, it is truly pivotal to comprehend the importance of additional large-scale studies with a longer follow-up to address gaps in this field; such as studies optimally conducted from the same center, from the same surgical team to directly compare the two methods and the diverse kinds of implants accessible for clinical use.

In conclusion, the surgical approach is a variable that might influence clinical and radiographic results in short and long term. RSA either with DP or AS approach has been confirmed to be an effective surgical procedure in terms of pain and function in rotator cuff tear arthropathies. The implication of RSA keeps on expanding as indications continue to outspread, and long-term results have been evaluated consistently. However, the prosthesis model, shoulder pathology, and experience of individual are at least equally critical comparing with a surgical approach in terms of the final outcome. In the ongoing debate among decision-makers about the two different eras of RSA, the answer is undoubtedly not easy at all. Both surgical approaches showed similar clinical outcomes; we believe that to a great extent is a decision of surgeon depending on the experience of an individual.

## Data Availability Statement

The original contributions presented in the study are included in the article/supplementary material, further inquiries can be directed to the corresponding author/s.

## Author Contributions

PG: contributed in the initial conception, data validation, formal analysis, and critical revision. AF: contributed in designing and drafting the manuscript. AV: contributed in the design and interpretation. GD: contributed in the interpretation and critical revision. EP: contributed in data curation, resources analysis, and drafting the manuscript. KT: contributed in the conception, interpretation, and critical revision. All authors provide their approval for the final version to be published.

## Conflict of Interest

The authors declare that the research was conducted in the absence of any commercial or financial relationships that could be construed as a potential conflict of interest.

## Publisher's Note

All claims expressed in this article are solely those of the authors and do not necessarily represent those of their affiliated organizations, or those of the publisher, the editors and the reviewers. Any product that may be evaluated in this article, or claim that may be made by its manufacturer, is not guaranteed or endorsed by the publisher.

## Abbreviations

AS: anterosuperior approach
DP: deltopectoral approach
RSA: reverse shoulder arthroplasty.
